# Detection of tick-borne bacterial DNA (*Rickettsia* sp.) in reptile ticks *Amblyomma moreliae* from New South Wales, Australia

**DOI:** 10.1007/s00436-023-08108-7

**Published:** 2024-01-09

**Authors:** Michelle Misong Kim, Glenn Shea, Jan Šlapeta

**Affiliations:** 1 Sydney School of Veterinary Science, Faculty of Science, The University of Sydney, Sydney, New South Wales 2006 Australia; 2 Australian Museum Research Institute, The Australian Museum, Sydney, Sydney, New South Wales 2006 Australia; 3 The University of Sydney Institute for Infectious Diseases, Sydney, New South Wales 2006 Australia

**Keywords:** *Amblyomma moreliae*, Snake, Lizard, Zoonosis, PCR

## Abstract

Ticks are major arthropod vectors of disease, transmitting tick-borne pathogens during blood meal episodes. *Rickettsia* spp. and *Borrelia* spp. are two tick-borne pathogens of zoonotic concern previously identified in DNA isolates from the tick genera *Amblyomma* and *Bothriocroton* associated with reptilian hosts in Australia. Some reports suggest that these reptile ticks bite and attach to humans via accidental parasitism and transmit disease, with the tick *Bothriocroton hydrosauri* known to transmit *Rickettsia honei* or Flinders Island Spotted Fever *Rickettsia* to humans. This descriptive study aims to identify the ticks collected from wild reptiles submitted to veterinary clinics and captured by snake rescuers from New South Wales (NSW), Australia, and detect the presence of tick-borne bacterial DNA using quantitative polymerase chain reaction (qPCR) to detect *Rickettsia* spp. and *Bartonella* spp. and conventional nested-PCR to detect *Borrelia* spp. Morphological identification revealed ticks removed from one eastern blue-tongued lizard (*Tiliqua scincoides scincoides*) from North-Eastern NSW (Lismore), one eastern blue-tongued lizard from the Greater Sydney area (Canley Heights), one diamond python (*Morelia spilota spilota*) from the Greater Sydney area (Woronora Heights) and one red-bellied black snake (*Pseudechis porphyriacus*) from the Greater Sydney Area (Cronulla) in New South Wales were *Amblyomma moreliae*. No ticks were positive for *Bartonella* spp. and *Borrelia* spp. DNA using real-time PCR targeting *ssr*A gene and nested PCR targeting *Borrelia*-specific 16S rRNA gene, respectively. Real-time PCR targeting *glt*A, *omp*A, *omp*B and 17kDa gene of *Rickettsia* spp. revealed 14 out of 16 ticks were positive. The undescribed *Rickettsia* sp. DNA was identical to that previously recovered from reptile ticks in Australia and closely related to *Rickettsia tamurae* and *Rickettsia monacensis,* both of which are aetiologic pathogens of the Spotted Fever Group Rickettsiosis (SFGR). These results accentuate the ongoing need for increased study efforts to understand zoonotic potential of bacteria from reptile ticks and the tick-reptile-human relationship.

**Supplementary Information:**

The online version contains supplementary material available at 10.1007/s00436-023-08108-7.

## Introduction

Ticks are major arthropod vectors of disease, with various vertebral hosts including humans. With the increasing global incidence of tick-borne disease, there is an emerging need to understand the tick-host relationship to mitigate the risks of such diseases impacting communities (Dantas-Torres et al. 2012). Rickettsioses and borrelioses are tick-borne diseases caused by obligate intracellular bacteria of the genus *Rickettsia*, and spirochaete bacteria of the genus *Borrelia*, respectively (Ostfeld and Keesing 2000; Whiley et al. 2016). Such tick-borne diseases include the Spotted Fever Group Rickettsiosis (SFGR) and Lyme Borreliosis (LB) which threaten the health and well-being of humans (Ostfeld and Keesing 2000; Whiley et al. 2016). In Australia, the main aetiological species of SFGR are *Rickettsia australis* and *Rickettsia honei* and there is no convincing evidence that Lyme disease nor the causative species *Borrelia burgdorferi* occurs in Australia (Collignon et al. 2016; Graves et al. 2006).

Some Australian reports demonstrate that there are *Amblyomma* and *Bothriocroton* species associated with reptile hosts which bite and attach on to humans, although such cases remain rare and noteworthy (Egan et al. 2022; Roberts 1970). Patients reported clinical presentations such as headaches, local cutaneous inflammation, tenderness of superficial inguinal lymph nodes, but no evidence of prolonged symptoms (Egan et al. 2022; Norval et al. 2020). Although further studies to screen the patient and the tick for the presence of tick-borne pathogens were not pursued, it is evident through these case reports that reptile ticks have the potential to parasitise humans.

The aim of this study was to detect the presence reptile-borne tick pathogen DNA in a small collection of ticks from reptiles from NSW, Australia. The study utilised quantitative PCR (qPCR) and nested PCR diagnostic assays to detect *Rickettsia* spp., *Bartonella* spp. and *Borrelia* spp. in ticks collected from wild reptiles submitted to veterinary clinics and captured by snake rescuers.

## Materials and methods

Between January 2022 to June 2022, reptile ticks (*n* = 16) acquired from reptiles (*n*=4) found across NSW, Australia were donated to us the University of Sydney by veterinary hospitals and snake catchers (Supplementary Table 1, Table 1). The ticks were stored in 70% (*w/v*) ethanol. All ticks were examined under stereomicroscope (5-200X Olympus, Macquarie Park, Australia) and digital microscope (Keyence, United States of America) for identification using published dichotomous keys and descriptions (Roberts 1970). Total DNA was isolated as previously described and stored at -20 °C (Chandra et al. 2022). A selection of ticks was processed for molecular identification using conventional PCR, targeting a ~650-nt fragment of cytochrome *c* oxidase subunit 1 (*cox*1*)* as previously described using S0725 and S0726 primers (Panetta et al. 2017). PCR products were sequenced (Macrogen Ltd., Seoul, South Korea) and sequences were assembled and compared to related *cox*1 sequences using CLC Main Workbench 22 (CLC bio, Qiagen, Chadstone, Australia). In addition, we assembled full *cox*1 from a single RNAseq dataset (SRR8074777) generated by Harvey et al. (2019) from 20 adults of *Amblyomma moreliae* collected from Blue-tongue lizard in Sydney, NSW.

**Table 1:** *Amblyomma moreliae* collected from reptiles in New South Wales, Australia

Reptile ID	Species	Locality (source)	Tick ID
MK-01	Eastern Blue-Tongue Lizard (*Tiliqua scincoides scincoides)*	North-East NSW, Lismore (veterinary clinic)	MK01-1; MK01-2
MK-02	Diamond Python (*Morelia spilota spilota)*	Greater Sydney, Woronora Heights (rescue)	MK02-1; MK02-2
MK-03	Red-Bellied Black Snake (*Pseudechis porphyriacus)*	Greater Sydney, Cronulla (rescue)	MK03-1; MK03-2
MK-04	Eastern Blue-Tongue Lizard (*Tiliqua scincoides scincoides)*	Greater Sydney, Canley Heights (veterinary clinic)	MK04-1; MK04-2; MK04-3; MK04-4; MK04-5; MK04-6; MK04-7; MK04-8; MK04-9; MK04-10

DNA from all sixteen ticks was screened using *Rickettsia* spp. / *Bartonella* spp. multiplex real-time PCR utilising Luna Universal Probe qPCR Master Mix (New England Biolabs, Victoria, Australia) targeting *glt*A citrate synthase gene (~75-bp amplicon) for *Rickettsia*, and a *ssr*A transfer-messenger RNA (~300-bp amplicon) for *Bartonella* as previously adopted (Diaz et al. 2012; Šlapeta and Šlapeta 2016; Stenos et al. 2005). Each run included a positive control carrying *Bartonella* and *Rickettsia* target DNA insert, a negative control with sterile PCR-grade water to control for contamination during PCR, and an additional blank template (NTC) to control for contamination during DNA extraction. The arbitrary real-time PCR threshold was set to a single threshold at 100rfu. Results were considered positive if samples yielded a C_t_ value <36 and negative if C_t_ > 40, and any samples that yielded C_t_ values ≥ 36 were suspect positive results (Huang et al. 2021).

Detection of *Borrelia* spp. spirochetes were performed using a 16S rRNA gene nested-PCR by amplifying a ~1250-nt fragment using previously published *Borrelia*-specific 16S rRNA primers as previously adopted (Loh et al. 2016). Each run included a blank negative control filled with sterile PCR-grade water. PCR was run on T100 Thermal Cycler (BioRad), as previously described (Panetta et al. 2017).

Ticks positive for rickettsial DNA were chosen for species identification using diagnostic conventional nested PCR assays targeting *glt*A gene, outer membrane protein A (*omp*A) and protein B (*omp*B) and surface 17kDa antigen (17kDa). A fragment (654 bp) of *glt*A was amplified using primer pair *gltA*-F1 (S0659) and *glt*A-R1 (S0660) followed by nested primer pair gltA-F2 (S0661) and gltA-R2 (S0662) (Šlapeta and Šlapeta 2016). Similarly, primer pairs ompARr190k71p (S1153) and ompARr190k.720n (S1154) followed by nested pair ompARr190k71p (S1153) and ompARr190k.602n (S1155); primer pair *omp*B-OF (S1158) and *omp*B-OR (S1159) followed by nested pair *omp*B-SFGIF (S1160) and *omp*B-SFGTGIR (S11601), and primer pair Rr17k.1p (S1150) and Rr17k.539n (S1151) followed by nested pair Rr17k.90p (S1152) and Rr17k.539n (S1151) were used to amplify *omp*A*, omp*B and 17kDa fragments respectively (Choi et al. 2005; Ishikura et al. 2003). Each PCR reaction included a negative controlled and was run on T100 Thermal Cycler (Biorad) as previously described (Šlapeta and Šlapeta 2016). Only positive samples of PCR amplicons were sequenced using amplification primers at Macrogen Inc. (Seoul, Korea).

**Fig. 1:**
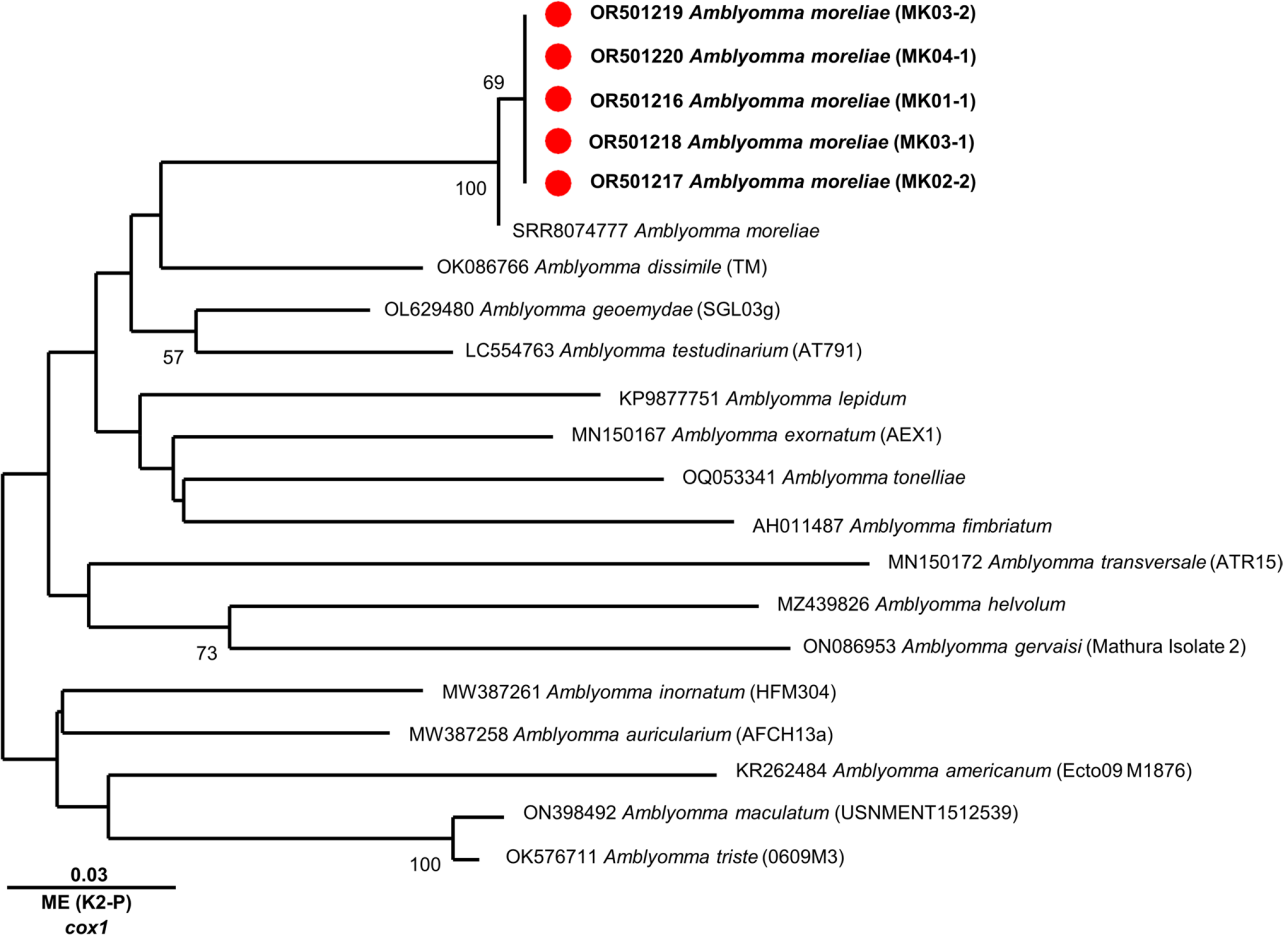
The evolutionary history of *Amblyomma cox*1 gene sequences. The tree was inferred using the Minimum Evolution (ME) method, with the evolutionary distances computed using the Kimura 2-parameter method conducted in MEGA11. The percentage of replicate trees in which the associated taxa clustered together in the bootstrap test (1,000 replicates) are shown next to the branches (>50%). All positions containing gaps and missing data were eliminated via complete deletion. There was a total of 222 positions in the final dataset. Evolutionary analyses were conducted in MEGA11. New *cox*1 sequences of *Amblyomma moreliae* (MK01-1, MK02-2, MK03-1, MK03-2, MK04-1) (Accession: OR501216 to OR501220) from this study are bolded with a red dot next to the name

**Fig. 2:**
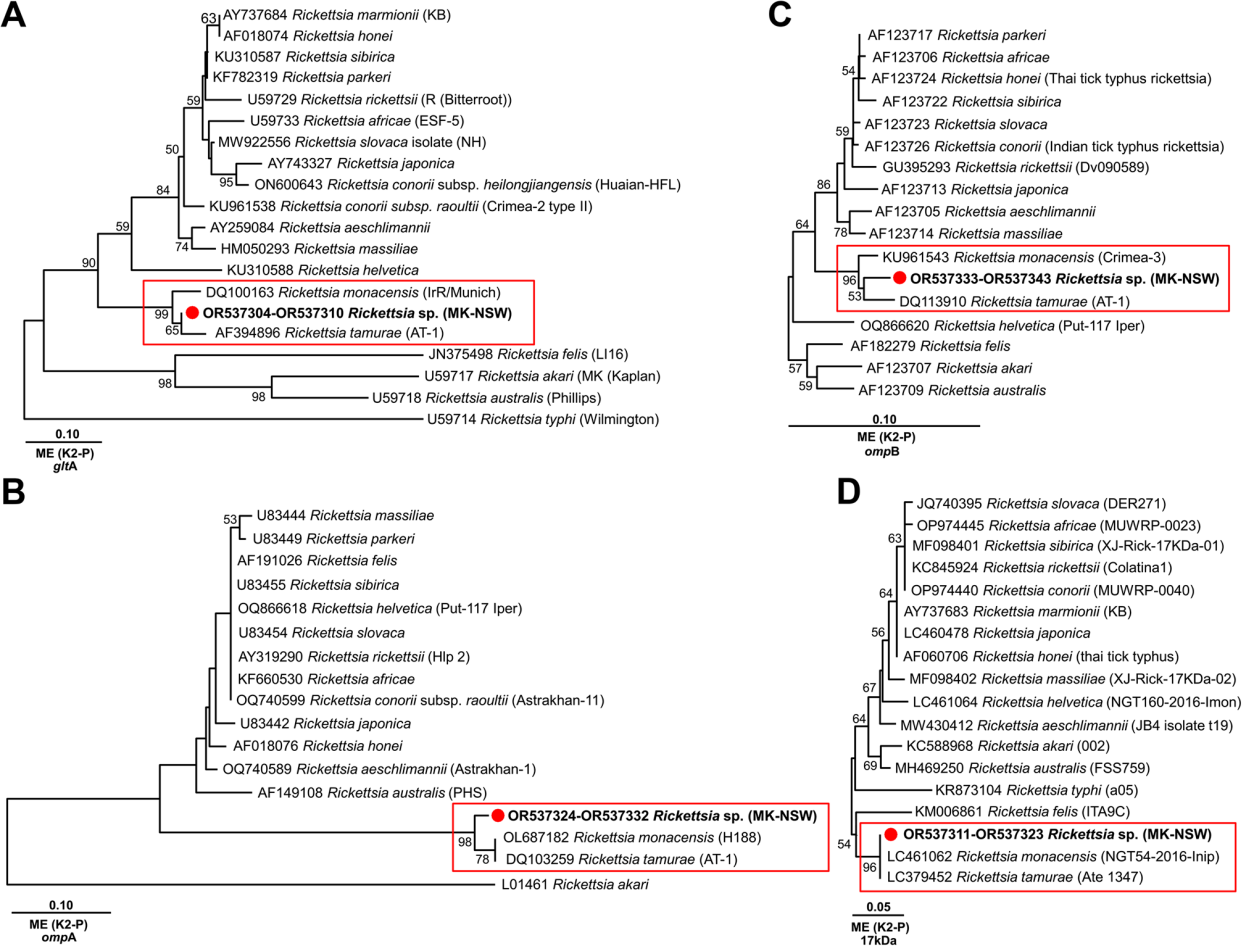
The evolutionary history of *Rickettsia glt*A, *omp*A, *omp*B and 17kDa gene sequences. The trees were inferred using the Minimum Evolution (ME) method, with the evolutionary distances computed using the Kimura 2-parameter method conducted in MEGA11. The percentage of replicate trees in which the associated taxa clustered together in the bootstrap test (1,000 replicates) are shown next to the branches (>50%). The sequenced highly similar to *Rickettsia* sp. sequenced in this study in a red box. (A) *glt*A gene sequences had a total of 606 positions in the final dataset. (B) *omp*A gene sequences had a total of 59 positions in the final dataset. (C) *omp*B gene sequences had a total of 309 positions in the final dataset. (D) 17kDa gene sequences had a total of 135 positions in the final dataset

Sequences were submitted to BLAST searches via BLASTN software (National Library to Medicine, Maryland, USA) and MEGA11 for phylogenetic comparison. Bootstrap replicates were obtained from 1,000 randomly generated alignments for manually constructed sequence assemblies for each for *cox*1, *glt*A, *omp*A, *omp*B, and 17kDa genes (Stecher et al. 2020). The evolutionary distances were computed using the Kimura 2-parameter method using available sequences for *Rickettsia* species as well as *Amblyomma* species. The generated matrices were used as basis for the formation of trees using the neighbour-joining minimum evolution method on MEGA11. Sequences generated were deposited to GenBank under the accession numbers OR501216–20, OR537304–43 (Supplementary Table S1, Figs. 1 and 2).

## Results

All ticks (*n* = 16) were morphologically identified as *A. moreliae* Koch, 1867. Ticks were removed from one eastern blue-tongued lizard (*Tiliqua scincoides scincoides*) from North-Eastern NSW (Lismore) (*n*=2), one eastern blue-tongued lizard from the Greater Sydney area (Canley Heights) (*n*=10), one diamond python (*Morelia spilota spilota*) from the Greater Sydney area (Woronora Heights) (*n*=2) and one red-bellied black snake (*Pseudechis porphyriacus*) from the Greater Sydney Area (Cronulla) (*n*=2) in New South Wales (Table 1). The c*ox*1 sequences of the five ticks sampled (MK01-1, MK02-2, MK03-1, MK03-2, MK04-1) were almost identical to each other (99.2–100%) and most similar to *Amblyomma testudinarium* Koch, 1844 (LC554763) (87.6%). At the time of publication, there were no DNA sequences of *A. moreliae* sequence in GenBank, but RNAseq data (SRR8074777) from *A. moreliae*. The assembled *cox*1 from RNAseq data was 99-99.7% identical across the PCR amplicons generated in this study. Multiple sequence alignment of *cox*1 and phylogenetic analysis with other *Amblyomma* shows *A. moreliae* shares a clade with *Amblyomma dissimile* Koch, 1844 (OK086766), which is nested within a larger clade with *A. testudinarium* (LC554763) and *A. geoemydae* Cantor, 1847 (OL629480) (Fig. 1).

The *Rickettsia* spp. and *Bartonella* spp. multiplex real-time PCR assay revealed the presence of *Rickettsia* spp. DNA in fourteen out of sixteen ticks with an average C_t_ value of 25.3 (min. 24.4, max. 35.5). Fourteen ticks DNA samples that were *Rickettsia* spp. DNA positive using the real-time PCR assay were characterised by multilocus typing using nested PCR at four *Rickettsia* spp. DNA loci. Seven tick samples yielded identical ~650-nt *Rickettsia glt*A sequences (OR537304–10), and multiple sequence alignment with phylogenetic analysis of *glt*A sequences from representative *Rickettsia* spp. revealed a high identity (100%) to six sequences of *R.* cf. *tamurae* from *Bothriocroton undatum* (Fabricius, 1775) (MG004673–78) from New South Wales, Australia (Panetta et al. 2017). Nine samples yielded identical ~488-nt *Rickettsia omp*A amplification products (OR537324–32) which revealed high identity (99.6%, 2/488) to a sequence from *R.* sp. 801a from *Amblyomma fimbriatum* Koch, 1844 (EU283837) from the Northern Territory, Australia (Vilcins et al. 2009). Eleven ticks yielded identical ~379-nt *omp*B products (OR537333–43) with 96% similarity to a sequence from *Rickettsia* sp. from *Ixodes boliviensis* Neumann, 1904 (MW699710). Thirteen ticks yielded identical ~410-nt 17kDa amplification products (OR537311–23) with high identity (100%) to a sequence from *Rickettsia* sp. 777c from *A. fimbriatum* (EU283838) from the Northern Territory, Australia (Vilcins et al. 2009). At least one tick from each of the four host reptiles were positive for *Rickettsia* spp. Sequence identity and phylogenetic position suggest that this is a novel species within the same clade as *Rickettsia tamurae* and *Rickettsia monacensis* (Fig. 2).

No ticks were positive for *Bartonella* spp. DNA (C_t_ value > 40) using the *Rickettsia* spp. and *Bartonella* spp. multiplex real-time PCR assay. DNA amplification of 16S rRNA using conventional nested PCR revealed no ticks positive for *Borrelia* DNA.

## Discussion

Ticks are amongst the most significant arthropod vectors of zoonotic disease, with a diverse pool of documented reptilian hosts. In Australia, *Amblyomma* and *Bothriocroton* are recognised as the main genera of ticks with reptiles documented as the main hosts for some of their species (Barker and Barker 2023). The identification of tick species is a fundamental task in the epidemiological studies of tick-born disease.

Here, the morphological identification enabled us to develop a molecular marker for future genetic identification and comparison to other related species (Mediannikov and Fenollar 2014). Key features which distinguish *A. moreliae* from other Australian reptile tick species, previously ascribed to the genera *Amblyomma* and *Aponomma*, are well-described in published dichotomous keys and descriptions (Roberts 1970). However, the generic taxonomy used by Roberts has subsequently been revised, with some species of *Aponomma* transferred to the new genus *Bothriocroton* Keirans, King & Sharrad, 1994, and the remaining *Aponomma* species, including the type species, transferred to *Amblyomma*, and thus the genus name *Aponomma* is no longer used (Barker and Murrell 2004; Klompen et al. 2002).

To the best of our knowledge, this study is the first to sequence *A. moreliae*. Currently, only *A. moreliae* transcriptomes (SRR8074777) are published and available (Harvey et al. 2019; Uribe et al. 2020). Whilst the purpose of the phylogenetic tree is to demonstrate the relationship between our Australian *A. moreliae* with other *Amblyomma* species, it only incorporates 1 of 2 Australasian *Amblyomma* species – *A. fimbriatum* – and none of the 11 other endemic Australian *Amblyomma* species (Barker and Barker 2023) (Fig. 1). Therefore, with the lack of endemic Australian *Amblyomma* representation in the phylogenetic tree, the relationship between *A. moreliae*, *A. dissimile, A. testudinarium* and *A. geoemydae* cannot be conclusive.

One notable diseases transmitted by ticks is SFGR, a zoonotic disease with a wide spectrum of severity ranging from fever, headache, eschar, rash, nausea, vomiting, to death even in young, healthy people (Mahajan 2012). The spotted fever rickettsial pathogens previously reported from Australia are *Rickettsia australis*, *Rickettsia honei* and *Rickettsia honei* subspecies *marmionii* (Graves et al. 2006; Stenos et al. 2003; Unsworth et al. 2007). In Australia, *R. honei* is the rickettsial species associated with both SFGR and reptile ticks but emerging studies are beginning to find evidence of novel rickettsial species closely related to *R. tamurae* (Panetta et al. 2017; Vilcins et al. 2009; Whiley et al. 2016). Similar to these studies, trees derived from analysis of the sequences of all four target genes (*glt*A, *omp*A, *omp*B, 17kDa) show that the novel rickettsial DNA sequenced from our ticks lies closely within the same clade as *R. tamurae* and *R. monacensis*, which are part of the SFGR documented to cause clinical rickettsial disease in East Asian and European countries, respectively (Chao et al. 2017; Dobler et al. 2009; Kim et al. 2010).

Although the outcome of this study is preliminary, the presence of rickettisal DNA closely related to SFGR pathogens adds to the growing number of species being found in Australia. This *Rickettisal* DNA appears to be indiscriminate against the reptilian host species involved in this study, thus warranting its potential to harbour in various reptiles. This warrants further efforts to characterise these rickettsiae and assess disease prevalence in a larger scale in Australian ticks and their hosts (Vilcins et al. 2009). Novel reptile-associated *Borrelia* spp. identified in *B. undatum*, *Amblyomma calabyi* Roberts, 1963, *A. fimbriatum*, and nymph *Amblyomma limbatum* Neumann, 1899 in Australia represents a distinct *Borrelia* clade which is evolutionarily, ecologically, and genetically unique to Lyme borreliosis and relapsing fever *Borrelia* groups (Gofton et al. 2023). The lack of *Borrelia* positive ticks in this study along with the lacking evidence of Lyme borreliosis and relapsing fever *Borrelia* groups and the existence of their ticks in Australia suggests that it is unlikely that Australian *A. moreliae* is an unlikely vector for *Borrelia*-related disease (Collignon et al. 2016; Jakab et al. 2022).

Using multilocus genotyping, we confirm the existence of an unnamed Australian species of *Rickettsia* which has previously been reported from the reptile ticks *A. fimbriatum* in the Northern Territory, in all stages of reptile ticks *Bothriocroton hydrosauri* (Denny, 1843) recovered from *Tiliqua rugosa* in South Australia, as well as *B. undatum* recovered from *Varanus varius* in New South Wales (Panetta et al. 2017; Vilcins et al. 2009; Whiley et al. 2016). We consider our recovered species a novel species that was previously labelled as *Rickettsia* cf. *tamurae* (MG004673 to MG004678) or *Rickettsia* sp. 777c from *A. fimbriatum* (EU283838) (Panetta et al. 2017; Vilcins et al. 2009). It will be important to establish an *in vitro* culture of this rickettsial bacterium before its formal description. Isolation and genomic characterisation will be required to confirm the species status of this novel yet widely distributed *Rickettsia* sp. Australia.

Our tick—*A. moreliae*—is an endemic Australian reptile tick species widespread throughout eastern states, NSW, Queensland, and Victoria, that is only occasionally reported from mammals (Barker and Barker 2023; Roberts 1964; Roberts 1970; Shea 1983). Its potential to transmit zoonotic tick-borne diseases is unknown, but evidence of it harbouring *Rickettsia* with high phylogenetic identity to species with known zoonotic potential and its presence in various reptilian species warrants further investigation to better understand the risks posed by reptilian ticks in Australia.

## Conclusion

In this study, utilised samples from reptiles in the Sydney area, were dominated by *A. moreliae*. Ticks in the sample tested positive for rickettsial DNA but not for *Bartonella* spp. and *Borrelia* spp. DNA. We confirm the existence of an unnamed Australian species of *Rickettsia* which has previously been originally reported from the reptile ticks in Australia. Future study efforts to identify the pathogenicity of this rickettsial pathogen can complement this study.

### Supplementary information

ESM 1 (XLSX 22 kb)

## Data Availability

All data included in the manuscript.
